# Functional brain networks for learning predictive statistics

**DOI:** 10.1016/j.cortex.2017.08.014

**Published:** 2018-10

**Authors:** Joseph Giorgio, Vasilis M. Karlaftis, Rui Wang, Yuan Shen, Peter Tino, Andrew Welchman, Zoe Kourtzi

**Affiliations:** a Department of Psychology, University of Cambridge, Cambridge, UK; b Key Laboratory of Mental Health, Institute of Psychology, Chinese Academy of Sciences, Beijing, China; c Department of Mathematical Sciences, Xi'an Jiaotong-Liverpool University, Suzhou, China; d School of Computer Science, University of Birmingham, Birmingham, UK

**Keywords:** Brain plasticity, fMRI, Functional Network Connectivity, Individual differences, Statistical learning

## Abstract

Making predictions about future events relies on interpreting streams of information that may initially appear incomprehensible. This skill relies on extracting regular patterns in space and time by mere exposure to the environment (i.e., without explicit feedback). Yet, we know little about the functional brain networks that mediate this type of statistical learning. Here, we test whether changes in the processing and connectivity of functional brain networks due to training relate to our ability to learn temporal regularities. By combining behavioral training and functional brain connectivity analysis, we demonstrate that individuals adapt to the environment's statistics as they change over time from simple repetition to probabilistic combinations. Further, we show that individual learning of temporal structures relates to decision strategy. Our fMRI results demonstrate that learning-dependent changes in fMRI activation within and functional connectivity between brain networks relate to individual variability in strategy. In particular, extracting the exact sequence statistics (i.e., matching) relates to changes in brain networks known to be involved in memory and stimulus-response associations, while selecting the most probable outcomes in a given context (i.e., maximizing) relates to changes in frontal and striatal networks. Thus, our findings provide evidence that dissociable brain networks mediate individual ability in learning behaviorally-relevant statistics.

## 1 Introduction

Successful interactions in a new environment entail interpreting initially incomprehensible streams of information and making predictions about upcoming events. The brain is thought to succeed in this challenge by finding regular patterns and meaningful structures that help us to predict and prepare for future actions. This skill is thought to rely on our ability to extract spatial and temporal regularities, often with minimal explicit feedback (Aslin and Newport, 2012, Perruchet and Pacton, 2006). For example, previous behavioral studies have shown that structured patterns become familiar after simple exposure to items (shapes, tones or syllables) that co-occur spatially or follow in a temporal sequence (Chun, 2000, Fiser and Aslin, 2002, Saffran et al., 1996, Saffran et al., 1999, Turk-Browne et al., 2005).

Functional imaging studies have identified key brain regions involved in the learning of statistical regularities. In particular, striatal and hippocampal regions have been implicated in the learning of temporal sequences (Aizenstein et al., 2004, Gheysen et al., 2011, Hsieh et al., 2014, Rauch et al., 1997, Rose et al., 2011, Schendan et al., 2003). Further, the medial temporal cortex has been implicated in learning of probabilistic associations (Schapiro et al., 2012, Turk-Browne et al., 2010). However, we know little about the functional brain networks and their interactions that mediate statistical learning of temporal structures.

Recent functional connectivity studies provide accumulating evidence for learning-dependent changes in human brain networks due to training in a range of tasks including visual perceptual learning (Baldassarre et al., 2012, Lewis et al., 2009), motor learning (Bassett et al., 2011, Ma et al., 2011, Sun et al., 2007), auditory learning (Ventura-Campos et al., 2013) and language learning (Veroude, Norris, Shumskaya, Gullberg, & Indefrey, 2010). These studies typically involve prolonged training with feedback. Here we ask whether mere exposure to streams of information (i.e., without trial-by-trial feedback) changes processing in functional brain networks that mediate our ability to extract statistical regularities.

**Fig. 1:**
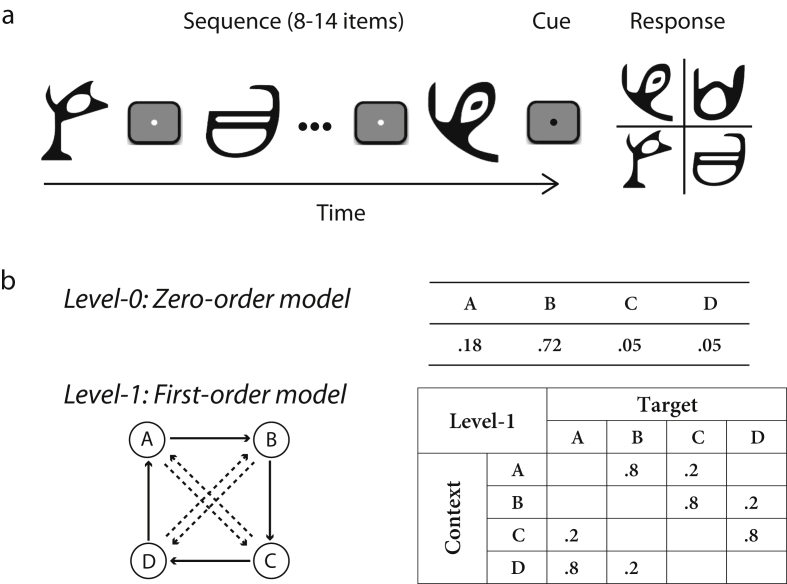
**Trial and sequence design**. (a) The trial design: 8–14 symbols were presented sequentially followed by a cue and the test display. (b) Sequence design: Markov models of the two context-length levels. For the zero-order model (level-0): different states (A, B, C, D) are assigned to four symbols with different probabilities. For the first-order model (level-1), diagrams indicate states (circles) and conditional probabilities (solid arrows: high probability; dashed arrows: low probability). Transitional probabilities are shown in a four-by-four (level-1) conditional probability matrix, where rows indicate the context and columns the corresponding target.

We combine behavioral measurements and multi-session fMRI (before and after training) to investigate processing in functional brain networks that mediate statistical learning of temporal structures. Event structures in the natural environment typically contain regularities at different scales from simple repetition to probabilistic combinations. To investigate the brain networks involved in extracting such structures unencumbered by past experience, we generated temporal sequences based on Markov models of different orders (i.e., context lengths of 0 or 1 previous item) (Fig. 1). We exposed participants to sequences of unfamiliar symbols and varied the sequence structure unbeknownst to the participants by increasing the context length. To facilitate learning, sequences were first determined by frequency statistics (i.e., occurrence probability per symbol), and then by context-based statistics (i.e., the probability of a given symbol appearing depends on the preceding symbol). Participants performed a prediction task, indicating which symbol they expected to appear next in the sequence. Following previous statistical learning paradigms, participants were exposed to the sequences without trial-by-trial feedback. We tested for improvement in the prediction task and fMRI activation changes in functional networks due to training (i.e., before *vs* after training on frequency and context-based statistics).

Further, we asked whether learning-dependent changes in functional brain networks relate to the participants' ability to learn temporal structures. Previous work (Acerbi et al., 2014, Eckstein et al., 2013, Erev and Barron, 2005, Lagnado et al., 2006, Murray et al., 2015, Shanks et al., 2002, Wozny et al., 2010) has highlighted the role of strategies in probabilistic learning and decision making and suggests that previous experience shapes the selection of decision strategies (Fulvio et al., 2014, Rieskamp and Otto, 2006). That is, observers are shown to match their choices stochastically according to the underlying input statistics or maximize their reward by selecting the most probable positively rewarded outcomes. Here, we tested whether learning-dependent changes in functional brain networks relate to the participants' decision strategy when learning frequency and context-based statistics.

Our behavioral results show that individuals adapt to the environment's statistics; that is, they are able to extract predictive structures that change over time. Further, we show that individual learning of structures relates to decision strategy; that is, individuals differed in their decision strategies, favoring probability maximization (i.e., extracting the most probable outcome in a given context) or matching the exact sequence statistics. We used this variability in decision strategy to interrogate fMRI activity in functional brain networks. Our results demonstrate that distinct brain networks mediate these two strategies. In particular, learning-dependent fMRI changes in functional brain networks relate to individual variability in decision strategy: matching relates to fMRI activation changes in brain networks involved in memory and stimulus-response associations (including Precuneus, Sensorimotor, Middle Temporal and the Right Central Executive), while maximizing relates to activation changes in frontal and striatal brain networks (including Basal Ganglia and the Left Central Executive). Further, increased functional connectivity due to training between networks involved in memory and stimulus-response associations relates to matching, while between frontal and striatal networks relates to maximization. Thus, our findings provide evidence for distinct functional brain networks that mediate individual ability to extract behaviorally-relevant statistics in variable environments.

## 2 Material and methods

### 2.1 Observers

Twenty-three participants (mean age = 21.8 years) were tested in multiple scanning and behavioral training sessions. The data from four participants were excluded from further imaging analysis due to excessive head movement. A single run from six of the remaining nineteen participants was also removed due to excessive head movement. All participants were naive to the aim of the study, had normal or corrected-to-normal vision and gave informed consent. This study was conducted in the School of Psychology, University of Birmingham and was approved by the University of Birmingham Ethics Committee.

### 2.2 Stimuli

Stimuli comprised four symbols chosen from Ndjuká syllabary (Fig. 1a). These symbols were highly discriminable from each other and were unfamiliar to the participants. Each symbol subtended 8.5° of visual angle and was presented in black on a mid-gray background. Experiments were controlled using Matlab and the Psychophysics toolbox 3 (Brainard, 1997, Pelli, 1997). For the behavioral training sessions, stimuli were presented on a 21-inch CRT monitor (ViewSonic P225f 1280 × 1024 pixel, 85 Hz frame rate) at a distance of 45 cm. For the pre and post-training fMRI scans, stimuli were presented using a projector and a mirror set-up (1280 × 1024 pixel, 60 Hz frame rate) at viewing distance of 67.5 cm. The physical size of the stimuli was adjusted so that angular size was constant during behavioral and scanning sessions.

### 2.3 Sequence design

To generate probabilistic sequences that differed in their structure, we used temporal Markov models and manipulated the memory order of the, which we refer to as the context length (Wang, Shen, Tino, Welchman, & Kourtzi, in press). The Markov model consists of a series of symbols, where the symbol at time *i* is determined probabilistically by the previous ‘*k*’ symbols. We refer to the symbol presented at time *i*, *s*(*i*), as the *target* and to the preceding *k*-tuple of symbols (*s*(*i−1*), *s*(*i−2*), …, *s*(*i−k*)) as the *context*. The value of ‘*k*’ is the order or level of the sequence:

*P*(*s*(*i*) |*s*(*i−1*), *s*(*i−2*), …, *s*(1)) = *P*(*s*(*i*) |*s*(*i−*1), *s*(*i−*2), …, *s*(*i−k*)), *k*< *i*

In our study, we used two levels of memory length; for *k* = *0,* *1*. The simplest *k* = *0th* order model is a memory-less source. This generates, at each time point *i*, a symbol according to symbol probability *P(s)*, without taking account of the previously generated symbols. The order *k* = *1* Markov model generates symbol *s*(*i*) at each time *i* conditional on the previously generated symbol *s*(*i−1*). This introduces a memory in the sequence; that is, the probability of a particular symbol at time *i* strongly depends on the preceding symbol *s*(*i−1*). Unconditional symbol probabilities *P*(*s*(*i*)) for the case *k* = *0* are replaced with conditional ones, *P*(*s*(*i*)|*s*(*i−1*)).

At each time point, the symbol that follows a given context is determined probabilistically, making the Markov sequences stochastic. The underlying Markov model can be represented through the associated context-conditional target probabilities. We used 4 symbols that we refer to as stimuli A, B, C and D. The correspondence between stimuli and symbols was counterbalanced across participants.

For level-0, the Markov model was based on the probability of symbol occurrence: one symbol had a high probability of occurrence, one low probability, while the remaining two symbols appeared rarely (Fig. 1b). For example, the probabilities of occurrence for the four symbols A, B, C and D were .18, .72, .05 and .05, respectively. Presentation of a given symbol was independent of the items that preceded it. For level-1, the target depended on the immediately preceding stimulus (Fig. 1b). Given a context (the last seen symbol), only one of two targets could follow; one had a high probability of being presented and the other a low probability (e.g., 80% *vs* 20%). For example, when Symbol A was presented, only symbols B or C were allowed to follow, and B had a higher probability of occurrence than C.

To test whether participants adapt to changes in the temporal structure, we ensured that the sequences across levels were matched for properties other than context-length. That is, sequences across levels were matched for the number of symbols presented (i.e., all four symbols were presented for both level-0 and level-1 sequences). To ensure that for level-1 participants learned context-target contingences rather than individual symbols, all symbols in level-1 were presented with equal frequency (i.e., marginal probability of each symbol was .25). These constraints resulted in differences in the probability distributions between level-0 and level-1. However, we designed the stochastic sources from which the sequences were generated so that the context-conditional uncertainty remained highly similar across levels. In particular, for the zero-order source, only two symbols were likely to occur most of the time; the remaining two symbols had very low probability (.05); this was introduced to ensure that there was no difference in the number of symbols presented across levels. Of the two dominant symbols, one was more probable (probability .72) than the other (probability .18). This structure is preserved in Markov chain of order 1, where conditional on the previous symbol, only two symbols were allowed to follow, one with higher probability (.80) than the other (.20). This ensures that the structure of the generated sequences across levels differed predominantly in memory order (i.e., context length) rather than context-conditional probability.

### 2.4 Procedure

Participants were initially familiarized with the task through a brief practice session (8 min) with random sequences (i.e., all four symbols were presented with equal probability 25% in random order). Following this, participants took part in multiple behavioral training and fMRI scanning sessions that were conducted on different days. Participants were trained with structured sequences and tested with both structured and random sequences to ensure that training was specific to the trained sequences.

In the first scanning session, participants were presented with zero- and first-order sequences and random sequences. Participants were then trained with zero-order sequences, and subsequently with first-order sequences. For each level, participants completed a minimum of 3 and a maximum of 5 training sessions (840–1400 trials). Training at each level ended when participants reached plateau performance (i.e., performance did not change significantly for two sessions). A post-training scanning session followed training per level (i.e., on the following day after completion of training) during which participants were presented with structured sequences determined by the statistics of the trained level and random sequences (90 trials each). The mean time interval (±standard deviation) between the pre-training and the post-training test sessions was 21.6 (±3.3) days.

### 2.5 Psychophysical training

Each training session comprised five blocks of structured sequences (56 trials per block) and lasted one hour. To ensure that sequences in each block were representative of the Markov model order per level, we generated 10,000 Markov sequences per level comprising 672 stimuli per sequence. We then estimated the Kullback–Leibler divergence (KL divergence) between each example sequence and the generating source. In particular, for level-0 sequences this was defined as:

$$KL=\sum_{target}Q\left(target\right)\;\log\left(\frac{Q(target)}{P(target)}\right)\text{,}$$

and for level-1 sequences this was defined as:

$$KL=\sum_{context}Q\left(context\right)\sum_{target}Q\left(taget|context\right)\;\log\left(\frac{Q(target|context)}{P(target|context)}\right)\text{,}$$

where *P( )* refers to probabilities or conditional probabilities derived from the presented sequences, and *Q( )* refers to those specified by the source. We selected fifty sequences with the lowest KL divergence (i.e., these sequences matched closely the Markov model per level). The sequences presented to the participants during the experiments were selected randomly from this sequence set.

For each trial, a sequence of 8–14 stimuli appeared in the center of the screen, one at a time in a continuous stream, each for 300 msec followed by a central white fixation dot (ISI) for 500 msec (Fig. 1a). This variable trial length ensured that participants maintained attention during the whole trial. Each block comprised equal number of trials with the same number of stimuli. The end of each sequence was indicated by a red dot cue that was presented for 500 msec. Following this, all four symbols were shown in a 2 × 2 grid. The positions of test stimuli were randomized from trial to trial. Participants were asked to indicate which symbol they expected to appear following the preceding sequence by pressing a key corresponding to the location of the predicted symbol. Participants learned a stimulus-key mapping during the familiarization phase: key ‘8’, ‘9’, ‘5’ and ‘6’ in the number pad corresponded to the four positions of the test stimuli – upper left, upper right, lower left and lower right, respectively. After the participant's response, a white circle appeared on the selected item for 300 msec to indicate the participant's choice, followed by a fixation dot for 150 msec (ITI) before the start of the next trial. If no response was made within 2 sec, a null response was recorded and the next trial started. Participants were given feedback (i.e., score in the form of performance index (PI), see Section 2.8) at the end of each block – rather than per-trial error feedback – that motivated them to continue with training.

### 2.6 Scanning sessions

The pre-training scanning session (Pre) included six runs (i.e., three runs per level) the order of which was randomized across participants. Scanning sessions after training per level (denoted as Post-0, Post-1) included nine runs of structured sequences determined by the same statistics as the corresponding trained level and random sequences*.* Each run comprised five blocks of structured and five blocks of random sequences that were presented in a random counterbalanced order (2 trials per blocks; a total of 10 structured and 10 random trials per run), with an additional two 16 sec fixation blocks, one at the beginning and one at the end of each run. The trial design was adjusted to afford modeling of fMRI signals within the scanning timing constraints. In particular, each trial comprised a sequence of 10 stimuli that were presented for 250 msec each, separated by a blank interval during which a white fixation dot was presented for 250 msec. Following the sequence, a response cue (central red dot) appeared on the screen for 4 sec before the test display (comprising four test stimuli) appeared for 1.5 sec. Participants were asked to indicate which symbol they expected to appear following the preceding sequence by pressing a key corresponding to the location of the predicted symbol. A white fixation was then presented for 5.5 sec before the start of the next trial. In contrast to the training sessions, no feedback was given during scanning.

### 2.7 fMRI data acquisition

The experiments were conducted at the Birmingham University Imaging Centre using a 3T Philips Achieva MRI scanner. T2*-weighted functional and T1-weighted anatomical (175 slices; 1 × 1 × 1 mm^3^ resolution) data were collected with a 32-channel head coil. Echo planar imaging (EPI) data (gradient echo-pulse sequences) were acquired from 32 slices (whole brain coverage; duration = 6 min; TR = 2 sec; TE = 35 msec; 2.5 × 2.5 × 4 mm^3^ resolution; SENSE).

### 2.8 Behavioral analysis

#### 2.8.1 Performance Index

We assessed participant responses in a probabilistic manner. We computed a Performance Index (PI) per context that quantifies the minimum overlap (min: minimum) between the distribution of participant responses and the distribution of presented targets estimated across 56 trials per block by:

$$\text{PI}\left(\text{context}\right)=\sum_{\text{target}}\min({\text{P}}_{\text{resp}}(\text{target}|\text{context}),\;{\text{P}}_{\text{pres}}(\text{target}|\text{context}))$$

where the sum is over targets from the symbol set A, B, C and D.

The overall PI is then computed as the average of the performance indices across contexts, PI (context), weighted by the corresponding stationary context probabilities:

$$\text{PI}=\sum_{\text{context}}\text{PI}\left(\text{context}\right)\cdot \text{P}\left(\text{context}\right)$$

To compare across different levels, we defined a normalized PI measure that quantifies participant performance relative to random guessing. We computed a random guess baseline; i.e., performance index PI_rand_ that reflects participant responses to targets with a) equal probability of 25% for each target per trial for level-0 (PI_rand_ = .53); b) equal probability for each target for a given context for level-1 (PI_rand_ = .45). To correct for differences in random-guess baselines across levels, we subtracted the random guess baseline from the performance index (PI_normalized_ = PI − PI_rand_).

#### 2.8.2 Strategy choice and strategy index

To quantify each participant's strategy, we compared individual participant response distributions (response-based model) to two baseline models: (i) probability matching, where probabilistic distributions are derived from the Markov models that generated the presented sequences (Model-matching) and (ii) a probability maximization model, where only the single most likely outcome is allowed for each context (Model-maximization). We used Kullback–Leibler (KL) divergence to compare the response distribution to each of these two models. KL is defined as follows:

$$KL=\sum_{target}M\left(target\right)\;\log\left(\frac{M(target)}{R(target)}\right)$$

for level-0 model, and

$$KL=\sum_{context}M\left(context\right)\sum_{target}M\left(target|context\right)\;\log\left(\frac{M(target|context)}{R\left(target|context\right)}\right)$$

for level-1 model, where *R( )* and *M( )* denote the probability distribution or conditional probability distribution derived from the human responses and the models (i.e., probability matching or maximization) respectively, across all the conditions.

We quantified the difference between the KL divergence from the response-based model to Model-matching and the KL divergence from the response-based model to Model-maximization. We refer to this quantity as strategy choice indicated by ΔKL (Model-maximization, Model-matching). We computed strategy choice per training block, resulting in a strategy curve across training for each individual participant. We then derived an individual strategy index by calculating the integral of each participant's strategy curve and subtracting it from the integral of the exact matching curve, as defined by Model-matching across training. We defined the integral curve difference (ICD) between individual strategy and exact matching as the individual strategy index. Negative strategy index indicates a strategy closer to matching, while positive index indicate a strategy closer to maximization.

### 2.9 fMRI data analysis

#### 2.9.1 Data pre-processing

We pre-processed the fMRI data in Matlab R2013a and SPM12 software package (http://www.fil.ion.ucl.ac.uk/spm/software/spm12/) following the pipeline described in recent work (Taylor et al., 2015). We first processed the T1 weighted anatomical images by applying brain extraction and segmentation. From the segmented T1 we created a white matter (WM) mask and a cerebrospinal fluid (CSF) mask. For each fMRI run, we corrected the EPI data for slice scan timing (i.e., to remove time shifts in slice acquisition) and motion (least squares correction). We co-registered each run to the T1 image and calculated the mean CSF and WM signal per volume. We then aligned the T1 image to MNI space (affine transformation) and applied the same transformation to the EPI data. Finally, we resliced the aligned EPI data to 3 × 3 × 4 mm^3^ resolution and applied spatial smoothing with a 5 mm isotropic FWHM Gaussian kernel.

#### 2.9.2 Independent component analysis (ICA)

We used group spatial ICA (GICA) (Calhoun and Adali, 2012, Calhoun et al., 2009, Haberecht et al., 2001, McKeown et al., 1998) to extract participant- and session-specific hemodynamic source locations using the Group ICA fMRI Toolbox (GIFT) (http://mialab.mrn.org/software/gift/). Pre-training sessions comprised 3 runs, whereas post-training sessions comprised 9 runs. To account for the difference in number of runs between sessions, we matched the post-training session runs to the pre-training session in their acquisition order and therefore, we included the matched 3 runs for subsequent analyses. Following pre-processing of each run, we performed intensity normalization and dimensionality reduction. We used the Minimum Description Length criteria (MDL) (Rissanen, 1978) to estimate the dimensionality and determine the number of independent components. We used a two-level dimensionality reduction procedure using PCA; first at the participant level and then at the group level. The ICA estimation was run 20 times and the component stability was estimated using ICASSO (Himberg, Hyvärinen, & Esposito, 2004).

This procedure resulted in 28 independent components. We generated participant- and session-specific spatial maps and timecourses for each component using GICA3 back reconstruction. Participant spatial maps were not scaled and, as intensity normalization was performed prior to ICA, they represent percent signal change. For further analysis, we extracted the timecourse per participant per component and regressed out the six motion parameters (translation and rotation) as well as the mean WM and CSF signal. We then removed slow drifts by applying linear detrending on the regressed timecourse (Van Dijk et al., 2010).

#### 2.9.3 Component selection

We used a quantitative method (Stevens, Kiehl, Pearlson, & Calhoun, 2007) to remove components of non-neuronal origin. We first converted each component's spatial map to a *z*-map and thresholded it at *z* = 1.96 to calculate its spatial correlation with gray matter (GM) and CSF probabilistic maps (as extracted from the MNI template). We rejected any component with a spatial correlation of R^2^ > .025 with CSF or WM and of R^2^ < .025 with GM. To supplement this method, we visually inspected all rejected components to verify that they were not of neuronal origin. This method resulted in 13 rejected components: 7 components had a high spatial correlation with CSF, 1 component had a high correlation with WM and 5 components had a low correlation with GM.

We labeled the selected components based on spatial correlation with known resting-state networks, as the brain's functional architecture at rest has been shown to relate to task-based networks (Fox and Raichle, 2007, Smith et al., 2009). We correlated the thresholded spatial maps with network templates (Allen et al., 2011) and labeled each component based on its highest correlation value to the network templates. In further analysis, we used only the selected components. To further denote the areas included in each selected component, we created participant-specific maps per component by averaging the maps across runs and sessions per participant. We then generated a group map based on one sample *t*-test on the participant average map (FWER corrected at *p* < .005). We visualized the significant clusters in xjView toolbox (http://www.alivelearn.net/xjview) and labeled them based on their peak voxels (Table 1).

**Table 1:** **ICA components**. Clusters within the 15 task-related components are extracted from the group maps and are organized into known functional groups (Allen et al., 2011). The table shows the number of voxels within each cluster, the *x*, *y*, *z* coordinates of the peak voxel in MNI space and the t-statistic of the peak voxel.

Network	Component	Areas	Voxels	*x*, *y*, *z* (mm)	t-Value
Attentional	CP 17	Inferior parietal R	387	48, −61, 42	23.91
		Cerebellum Posterior L	151	−12, −73, −34	18.51
		Inferior frontal gyrus R	817	45, 41, 14	16.35
		Thalamus R	67	9, −22, 6	15.95
		Putamen R	155	30, 14, −6	14.96
		Inferior parietal L	57	−48, −46, 50	11.75
	CP 21	Inferior parietal L	414	−36, −58, 42	16.32
		Cerebellum Posterior R	147	21, −67, −34	15.29
		Middle frontal gyrus L	658	−45, 23, 30	15.04
		Putamen L	46	−33, −16, −6	14.08
		Insula L	25	−27, 17, −2	11.76
	CP 24	Cingulate Gyrus BL	3742	6, 20, 38	28.71
		Cerebellum Posterior R	23	36, −67, −26	10.32
Basal Ganglia	CP 13	Caudate R/L	1548	18, 17, 2	28.46
	CP 27	Putamen R/L	1321	−24, 2, −6	25.81
		Cingulate Gyrus BL	86	6, −1, 46	12.07
		Cerebellum Anterior L	20	−3, −58, −38	11.17
Default mode	CP 20	Precuneus R/L	819	12, −67, 30	21.61
		Cingulate R/L	251	12, 32, 18	15.51
		Superior Frontal Gyrus L	26	−24, 41, 22	11.86
		Inferior parietal R	20	48, −55, 42	10.72
	CP 23	Anterior Cingulate R/L	1834	−6, 44, 10	24.39
		Posterior Cingulate R/L	121	−3, −46, 30	18.83
		Cingulate gyrus L	32	−3, −16, 38	11.45
		Superior Temporal Gyrus L	68	−48, −58, 26	12.18
		Cerebellum Posterior R	52	27, −79, −30	11.02
		Superior Temporal Gyrus R	26	54, −61, 38	10.61
		Putamen R	24	30, 8, 2	10.48
	CP 26	Precuneus R/L	1011	6, −61, 18	21.88
		Middle Temporal Gyrus R	237	45, −64, 22	21.75
		Middle Temporal Gyrus L	233	−48, −67, 14	13.61
		Postcentral Gyrus R	93	48, 8, 26	14.71
Sensorimotor	CP 5	Superior Temporal Gyrus L	516	−45, −19, 6	19.38
		Superior Temporal Gyrus R	654	48, −10, 6	17.27
		Middle frontal gyrus L	24	−3, −1, 62	12.41
	CP 6	Postcentral Gyrus L	448	−42, −28, 54	25.15
		Precentral Gyrus R	91	57, −13, 34	14.48
		Cerebellum Anterior R	72	21, −52, −26	12.48
		Postcentral Gyrus R	30	36, −7, 62	10.96
		Parietal Superior L	20	−21, −61, 58	10.03
	CP 10	Paracentral R/L	1653	21, −31, 66	24.7
		Cerebellum Anterior L	125	−6, −46, −18	12.75
	CP 19	Insula L	139	−39, −13, −2	17.42
		Supramarginal R	167	57, −28, 26	17.17
		Insula R	177	42, −10, −6	16.19
		Supramarginal L	114	−63, −31, 22	15.48
		Cingulate Gyrus R/L	87	12, −34, 38	13.3
		Precuneus R/L	33	−6, −49, 58	13.21
		Postcentral R	20	21, −46, 66	11.2
		Middle Temporal Gyrus L	22	−54, −61, 6	9.83
Visual	CP 7	Lingual Gyrus R/L	1197	5, −63, 2	31.77
		Cerebellum Declive BL	47	−3, −73, −26	15.43
	CP 12	Middle Occipital Gyrus R/L	1730	30, −85, 18	22.85
		Posterior Cingulate R/L	107	1, −31, 26	15.94
Cerebellum	CP 16	Cerebellum Anterior Lobe BL	3013	30, −58, −34	36.86
		Precuneus R/L	30	3, −58, 38	10.51

#### 2.9.4 GLM-based analysis

We generated a GLM event-related (epoch) design and ran a multiple regression analysis on each component's timecourse (treated for nuisance variables: 6-motion parameters, CSF and WM) per participant per run. The GLM design was composed of random and structured trial blocks convolved with the hemodynamic response function. The output of the regression is a set of β weights (i.e., parameter estimates) for the task conditions (random, structured sequences); where the β weights represent the degree to which the component timecourse is modulated by each task condition. We then averaged the β weights of each task condition across runs resulting in a single value for each condition per participant per component per session.

To test whether component activation changes in relation to individual behavior (i.e., strategy), we correlated strategy index for frequency (level-0) and context-based (level-1) statistics with change of β weight (i.e., post minus pre-training) per component, separately for each task condition (random, structured). We used the Robust Correlation Toolbox (Pernet, Wilcox, & Rousselet, 2013) and Pearson's skipped correlation to account for potential outliers. We accepted as significant the correlations where the bootstrapped 95% confidence interval (CI) after 1000 permutations doesn't cross the zero origin.

#### 2.9.5 Functional Network Connectivity (FNC)

To investigate the functional interaction of the networks identified in the GLM-based analysis we calculated the between network connectivity of these components (Jafri, Pearlson, Stevens, & Calhoun, 2008). We defined as FNC the correlation of each component's timecourse (after nuisance regression and detrending) with every other component's timecourse, per participant. We converted the correlation coefficients to *z*-scores (Fisher *z*-transform) and averaged the values across runs for each pair of components; deriving one connectivity value per participant per session. We then correlated the change in average *z*-score (post minus pre-training) with strategy for frequency (level-0) and context-based (level-1) statistics using the Robust Correlation method.

## 3 Results

### 3.1 Behavioral performance

Previous studies have compared learning of different spatiotemporal contingencies in separate experiments across different participant groups (Fiser and Aslin, 2002, Fiser and Aslin, 2005). Here, to investigate whether individuals extract changes in structure, we presented the same participants with sequences that changed in structure unbeknownst to them (Fig. 1a). We parameterized sequence structure based on the memory-order of the Markov models used to generate the sequences (see Section 2.3); that is, the degree to which the presentation of a symbol depended on the history of previously presented symbols (Fig. 1b). We first presented participants with simple zero-order sequences (level-0) followed by more complex first-order sequences (level-1), as previous work has shown that temporal dependencies are more difficult to learn as their length increases (van den Bos & Poletiek, 2008) and training with simple dependencies may facilitate learning of more complex contingencies (Antoniou, Ettlinger, & Wong, 2016). Zero-order sequences (level-0) were context-less; that is, the presentation of each symbol depended only on the probability of occurrence of each symbol. For first-order sequences (level-1), the presentation of a particular symbol was conditionally dependent on the previously presented symbol (i.e., context length of one).

As the sequences we employed were probabilistic, we developed a probabilistic measure to assess participants' performance in the prediction task. Specifically, we computed a PI that indicates how closely the probability distribution of the participant responses matched the probability distribution of the presented symbols. This is preferable to a simple measure of accuracy because the probabilistic nature of the sequences means that the ‘correct’ upcoming symbol is not uniquely specified; thus, designating a particular choice as correct or incorrect is often arbitrary.

**Fig. 2:**
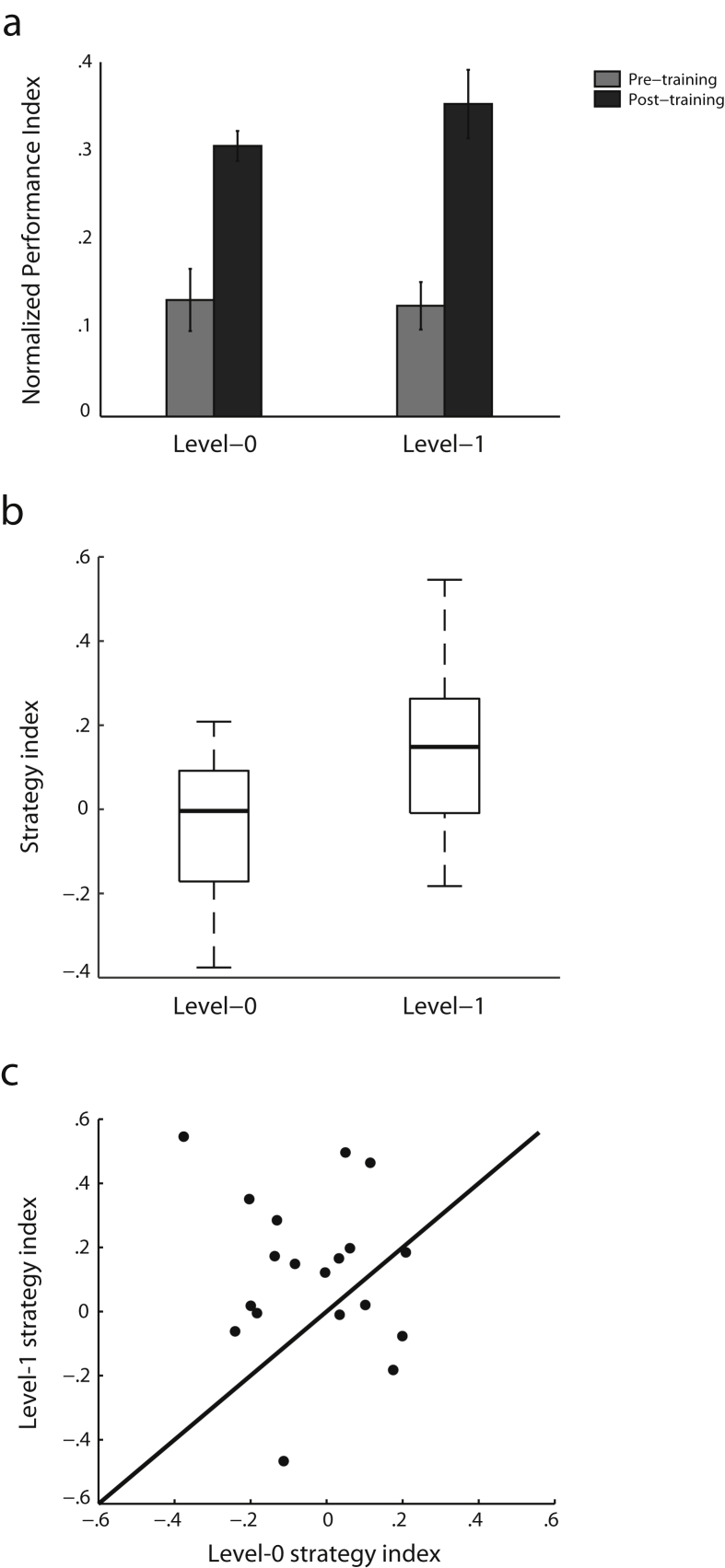
**Behavioral performance**. (a) Mean normalized performance index (PI) across participants per level during pre-training (gray bars) and post-training (black bars) test sessions. Error bars indicate standard error of the mean across participants. (b) Strategy index boxplots for level-0 and level-1 indicate individual variability. The upper and lower error bars display the minimum and maximum data values and the central boxes represent the interquartile range (25th to 75th percentiles). The thick line in the central boxes represents the median. (c) Scatterplot of strategy index for level-0 against strategy index for level-1.

Comparing normalized performance (i.e., after subtracting performance based on random guessing) before and after training per level (Fig. 2a) showed that participants improved substantially in learning probabilistic structures. A two-way repeated measures ANOVA with Session (Pre, Post) and Level (level-0, level-1) showed a significant main effect of Session [*F*(1,18) = 58.7, *p* < .001], but no significant effect of Level [*F*(1,18) = .6, *p* = .459] nor a significant interaction [*F*(1,18) = .6, *p* = .459], indicating that participants improved similarly at both levels through training. Further, we asked whether these learning effects were specific to the trained structured sequences. We contrasted performance on structured versus random sequences before and after training sessions. A repeated-measures ANOVA showed a significant interaction of Session (Pre, Post) and Sequence type (structured, random) for level-0 [*F*(1,18) = 20.5, *p* < .001] and level-1 [*F*(1,18) = 58.6, *p* < .001], suggesting that learning improvement was specific to the structured sequences.

### 3.2 Decision strategies: matching versus maximization

Previous work (Acerbi et al., 2014, Eckstein et al., 2013, Fulvio et al., 2014, Lagnado et al., 2006, Murray et al., 2015, Rieskamp and Otto, 2006, Shanks et al., 2002) on probabilistic learning and decision making has proposed that individuals use two possible decision strategies when making a choice: matching versus maximization. Observers have been shown to either match their choices stochastically according to the underlying input statistics or to maximize their reward by selecting the most probable positively rewarded outcomes. In the context of our task, as the Markov models that generated stimulus sequences were stochastic, participants needed to learn the probabilities of different outcomes to succeed in the prediction task. It is possible that participants used probability maximization whereby they always select the most probable outcome in a particular context. Alternatively, participants might learn the relative probabilities of each symbol [e.g., *p*(A) = .18; *p*(B) = .72, *p*(C) = .05; *p*(D) = .05] and respond so as to reproduce this distribution, a strategy referred to as probability matching.

To quantify participants' strategies across training, we computed a strategy index that indicates each participant's preference (on a continuous scale) for responding using probability matching versus maximization (Fig. 2b). Fig. 2b and c indicate variability in strategy index across participants. Comparing individual strategy across levels showed significantly higher values for level-1 compared to level-0 [t(18) = 2.2, *p* = .04], suggesting that participants adopted a strategy closer to maximization when learning context-based rather than frequency statistics (Fig. 2c). Note, that this relationship was not confounded by differences in performance, as there were no significant correlations (level-0: *r* = .34, *p* = .19; level-1: *r* = .04, *p* = .88) between performance after training and strategy index. Further, we conducted two additional analyses to control for the possibility that the differences we observed in strategy index between levels may be confounded by differences in the probability distributions between levels (i.e., 72% *vs* 80% probability for the most frequent target for level-0 *vs* level-1) and PI. First, we observed significantly higher strategy index for level-1 compared to level-0 [t(18) = 2.19, *p* = .042] after scaling the strategy index in level-0 by .8/.72 (i.e., the ratio of maximum PI for exact maximization for level-1 *vs* level-0). Second, strategy index remained higher for level-1 than level-0 [t(18) = 2.36, *p* = 0.030] after regressing out the post-training PI from strategy index per level. Thus, our result showing higher strategy index for level-1 than level-0 is unlikely to be confounded by differences in PI or the probability distributions between levels.

Finally, participants were exposed to the sequences without trial-by-trial feedback, but were given block feedback about their performance that motivated them to continue with training. A control experiment during which the participants were not given any feedback showed similar results to our main experiment; that is, higher strategy index for level-1 than level-0, suggesting that differences in the strategy between levels could not be simply attributed to feedback. Taken together, these results suggest that participants adopt a strategy closer to maximization for learning higher-order sequences (i.e., context-based statistics) than simple frequency statistics. This is consistent with previous studies showing that participants adopt a strategy closer to matching when learning a simple probabilistic task in the absence of trial-by-trial feedback (Shanks et al., 2002). However, for more complex probabilistic tasks, participants weight their responses towards the most likely outcome (i.e., adopt a strategy closer to maximization) after training (Lagnado et al., 2006).

### 3.3 fMRI analysis: functional brain networks

**Fig. 3:**
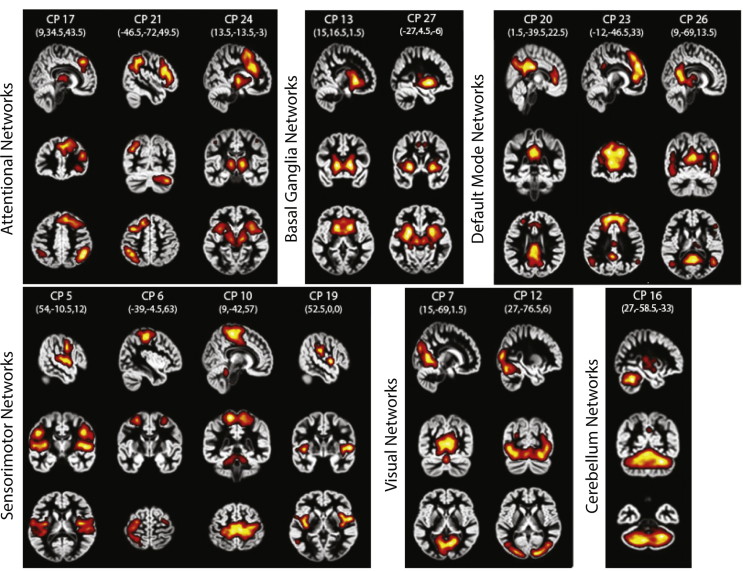
**Spatial maps of ICA task-related components**. 15 task-related components are shown organized into known functional groups (Allen et al., 2011). Spatial maps are thresholded at *p* < .005 (FWER corrected) and displayed in neurological convention (left is left) on the MNI template. The *x*, *y*, *z* coordinates per component denote the location of the sagittal, coronal and axial slices, respectively.

To identify functional brain networks that mediate our ability to adapt to changes in temporal statistics, we performed fMRI on participants before and after training on each level with structured and random sequences. First, we decomposed the fMRI timecourse into functionally connected components (i.e., components comprising voxel clusters with correlated fMRI time course) using ICA and selected components of neuronal origin using a spatial correlation method with known brain networks (Allen et al., 2011) (Fig. 3, Table 1). We then tested whether learning-dependent changes in fMRI activation in these brain networks relate to individual strategy when learning frequency and context-based statistics. For each component we extracted a β weight across voxels for structured and random sequences per session (pre-, post-training). We correlated learning-dependent changes in fMRI signal (post-pre β weight) for structured sequences with individual strategy. Positive correlations indicate increased activation after training that relates to maximization, while negative correlations indicate increased activation that relates to matching, as negative strategy index indicates strategy towards matching.

**Fig. 4:**
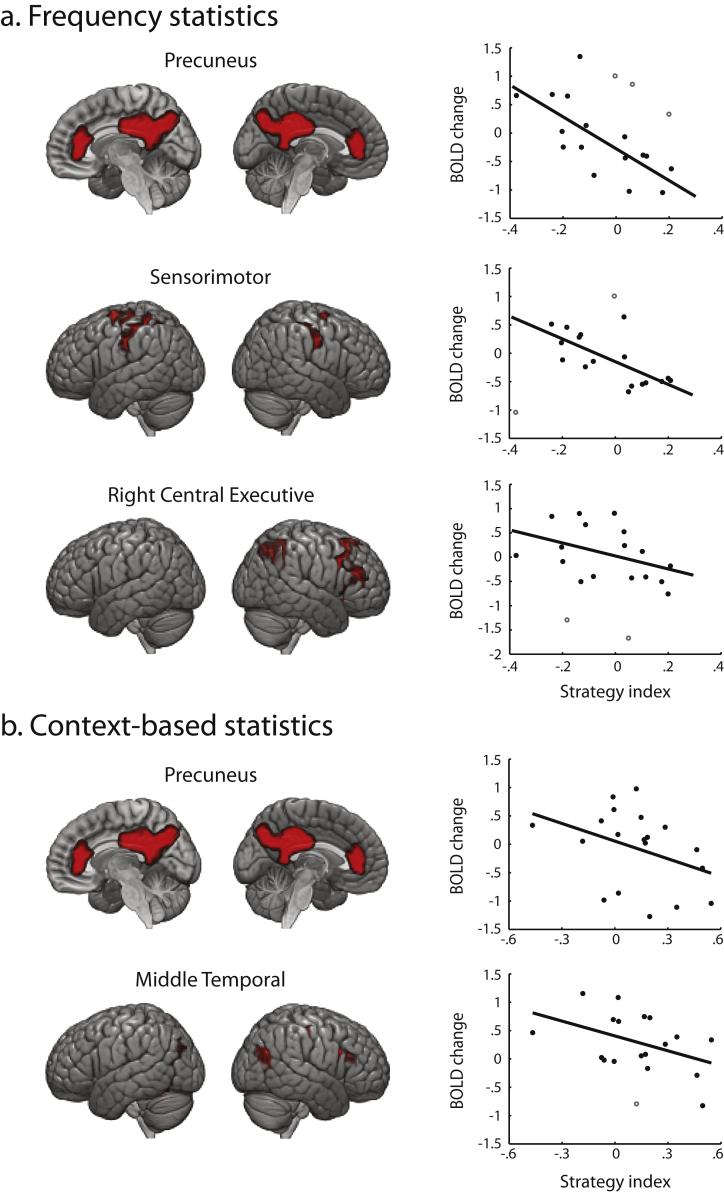
**ICA components related to matching strategy**. Average spatial maps showing significant negative correlation of BOLD change (post minus pre-training) with strategy index for (a) Learning frequency statistics: Precuneus, Sensorimotor and Right Central Executive. (b) Learning context-based statistics: Precuneus and Middle Temporal. Spatial maps are averaged across sessions, thresholded at *p* < .005 (FWER corrected) and displayed in neurological convention (left is left) on the MNI template. Open circles in the correlation plots denote outliers.

First, we observed significant negative correlations between learning-dependent fMRI changes and strategy index in functional brain networks known to be involved in memory processes and stimulus-response associations. In particular, for learning frequency statistics (Fig. 4a), we found significant negative correlations of fMRI activation change in the Precuneus (CP_20, peak activations in bilateral Precuneus and cingulate; *r* = −.70, CI = [−.88, −.48]), the Sensorimotor (CP_6, peak activations in bilateral precentral and postcentral gyri; *r* = −.70, CI = [−.90, −.42]) and the Right Central Executive (CP_17, peak activations in right inferior parietal and right inferior frontal gyrus; *r* = −.42, CI = [−.73, −.07]) networks with strategy. For learning context-based statistics (Fig. 4b), we found significant negative correlations of fMRI activation change in the Precuneus (CP_20; *r* = −.37, CI = [−.68, −.03]) and the Middle Temporal (CP_26, peak activations in bilateral Precuneus and Middle Temporal gyrus extending medially into parahippocampal cortex; *r* = −.44, CI = [−.74, −.01]) networks with strategy. These results suggest that increased functional activation in these brain networks after training relates to matching the exact sequence statistics. This is consistent with the role of Precuneus and cingulate in memory retrieval (Wagner et al., 2005, Cabeza et al., 2008, St. Jacques et al., 2011) in the context of episodic and working memory tasks (Nyberg, Forkstam, Petersson, Cabeza, & Ingvar, 2002). Further, Sensorimotor areas have been implicated in the consolidation of stimulus-response associations, mainly at early stages of motor consolidation (Muellbacher et al., 2002). Similarly, the Right Central Executive Network has been implicated in the initial stages of learning (Seger et al., 2000). Thus, these networks contribute at the initial training on frequency statistics, while the Middle Temporal network contributes at later learning of context-based statistics, as this brain network has been implicated in episodic memory and mnemonic tasks involving longer memory length (Cabeza et al., 2008, Nyberg et al., 2002, Vincent et al., 2006).

**Fig. 5:**
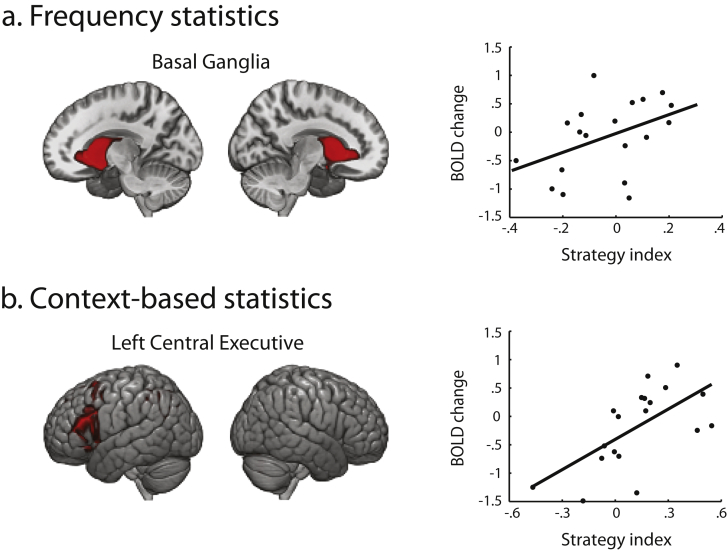
**ICA components related to maximization strategy**. Average spatial maps showing significant positive correlation of BOLD change (post minus pre-training) with strategy index for: (a) Learning frequency statistics: Basal Ganglia. (b) Learning context-based statistics: Left Central Executive. Spatial maps are averaged across sessions, thresholded at *p* < .005 (FWER corrected) and displayed in neurological convention (left is left) on the MNI template.

In contrast, we observed significant positive correlations between learning-dependent fMRI changes and strategy in the Basal Ganglia and the Left Central Executive Networks. In particular, for learning frequency statistics, we found a significant positive correlation of fMRI activation change in the Basal Ganglia Network (CP_13, peak activation in bilateral caudate) with strategy (*r* = .43, CI = [.04, .72]) (Fig. 5a), suggesting involvement of Basal Ganglia in learning by maximizing. This is consistent with previous work suggesting that Basal Ganglia is involved in the consolidation of the stimulus-response mapping (Albouy et al., 2008, Shohamy et al., 2008) and category learning (Ashby and Maddox, 2005, Seger and Cincotta, 2005). In particular, previous work on humans and animals emphasizes the role of the caudate in switching between strategies (Cools et al., 2004, Monchi et al., 2001, Seger and Cincotta, 2005), and learning after a rule reversal (Cools et al., 2002, Pasupathy and Miller, 2005). For learning context-based statistics, we found a significant positive correlation of fMRI activation change in the Left Central Executive Network (CP_21, peak activations in left inferior parietal and left middle frontal gyrus) with strategy (*r* = .63, CI = [.29, .84]) (Fig. 5b), suggesting that higher activation after training in this region relates to maximization. Executive networks have been implicated in holding and updating task rules (Ridderinkhof et al., 2004, Vincent et al., 2008, D'Ardenne et al., 2012). In particular, increased activation in the Left Central Executive Network has been shown after training in the context of category learning (Seger et al., 2000). This is consistent with our behavioral results showing that participants adopt a stronger maximization strategy during later training on context-based statistics.

Finally, we tested whether our results were specific to the learned structured sequences. We computed fMRI activation for random sequences in brain networks that showed significant correlations with strategy for structured sequences. For frequency statistics, fMRI activation change in the Precuneus Network showed a significant negative correlation with strategy (*r* = −.53, CI = [−.81, −.11]). For context-based statistics: a) activation change in the Middle Temporal Network (*r* = −.59, CI = [−.87, −.14]) and the Precuneus Network (*r* = −.57, CI = [−.81, −.25]) showed a significant negative correlation with strategy b) activation change in the Left Central Executive Network showed a significant positive correlation with strategy (*r* = .61, CI = [.25, .79]). To compare correlations for structured versus random sequences, we used Steiger *z*-score comparison (Lee & Preacher, 2013), for comparison of dependent correlations with a shared variable (i.e., strategy index). We found significantly higher negative correlations for structured versus random trials in: a) Precuneus (*z* = −2.19, *p* = .029), b) Right Central Executive (*z* = −2.43, *p* = .015) and c) Sensorimotor (*z* = −2.92, *p* = .004) Networks. These results suggest differences in the processing of structured versus random sequences primarily when participants learn by matching, as this strategy requires learning the exact sequence statistics that differ between these two sequence types.

### 3.4 Functional Network Connectivity (FNC)

**Fig. 6:**
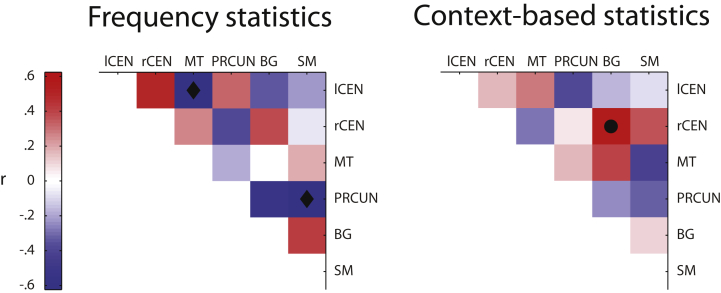
**Functional Network Connectivity (FNC) change related to strategy**. Correlation matrix of FNC change (post minus pre-training) with strategy index for: (a) frequency statistics and (b) context-based statistics. Black dots indicate significant positive, while black diamonds significant negative correlations (at 95% bootstrapped confidence intervals) of FNC change with strategy index. ICA components included in this analysis are: Left Central Executive Network (lCEN), Right Central Executive Network (rCEN), Middle Temporal (MT), Precuneus (PRCUN), Basal Ganglia (BG) and Sensorimotor (SM).

Our analyses so far identified brain networks that show learning-dependent changes in functional processing that relate to individual strategy for learning temporal structures. Next, we asked whether learning-dependent changes in the connectivity between these networks relate to individual strategy when learning frequency and context-based statistics. We calculated pairwise correlations between the six brain networks (Precuneus, Sensorimotor, Right Central Executive, Middle Temporal, Basal Ganglia, Left Central Executive) that showed significant correlations with strategy (see Section 3.3). We calculated these correlations for each session (Pre, Post-0, Post-1) and converted them to *z*-scores (Fisher *z*). We then correlated change (post minus pre-training *z*-score) in FNC with strategy index to assess the relationship of strategy with changes in between-network connectivity (Fig. 6).

For frequency statistics, we found that a) connectivity change between Left Central Executive and Middle Temporal Networks correlated negatively with strategy (*r* = −.62, CI = [−.86, −.18]), and b) connectivity change between Precuneus and Sensorimotor Networks correlated negatively with strategy (*r* = −.62, CI = [−.88, −.15]). These results suggest that increased connectivity between these networks with training relates to learning by matching the exact sequence statistics. For context-based statistics, we found that connectivity change between Right Central Executive and Basal Ganglia Networks correlated positively with strategy (*r* = .55, CI = [.01, .85]), suggesting that increased connectivity between these networks with training relates to maximization. These results are consistent with previous work highlighting the role of Central Executive Networks in controlling learning of contextual and stimulus-response associations (Ridderinkhof et al., 2004, D'Ardenne et al., 2012). Further, recent neurophysiology findings (Antzoulatos & Miller, 2014) show enhanced connectivity between prefrontal cortex and Basal Ganglia in the context of category learning, suggesting that fast learning in the Basal Ganglia may train slower learning in the frontal cortex that may facilitate the generalization and abstraction of learned associations.

This functional connectivity analysis is consistent with our previous analyses showing fronto-striatal networks involved in maximization, the strategy for which participants showed stronger preference when learning context-based statistics (Fig. 2b and c). Our results provide complementary evidence that learning-dependent changes in the connectivity of brain networks known to be involved in memory and stimulus-response associations mediate learning by matching the exact sequence statistics, while connectivity changes in frontal and striatal networks mediate learning by maximizing (i.e., extracting the most probable outcome in a given context).

## 4 Discussion

Here, we investigate the functional brain networks that mediate our ability to adapt to changes in the environment's statistics and make predictions. Our behavioral results demonstrate that individuals adapt to changes in temporal structure and extract the relevant frequency or context-based statistics for making predictions of upcoming events. Our fMRI results provide evidence for dissociated functional brain networks that mediate our ability to extract behaviorally-relevant statistics.

Our modeling approach allows us to track participants' predictions and their strategies during training. We demonstrate that learning predictive structures relates to individual variability in decision strategies: that is, individuals favored either probability maximization (i.e., extracting the most probable outcome in a given context) or matching the exact sequence statistics. Previous behavioral studies have reported individual variability in decision strategy in the context of probabilistic learning tasks and suggested that strategies change during the course of training with feedback (Gluck et al., 2002, Lagnado et al., 2006, Shanks et al., 2002). Here we show that decision strategy relates to sequence structure; that is, learning context-based statistics relates to stronger maximization than learning simple frequency statistics. Further, we provide evidence that these decision strategies engage distinct functional brain networks: matching relates to changes in fMRI activation within and functional connectivity between brain networks involved in memory and stimulus-response associations, while maximizing relates to changes in frontal and striatal brain networks.

Previous work has implicated these brain networks in reinforcement learning [e.g., for reviews (Robbins, 2007, Balleine and O'Doherty, 2010)]. Previous brain imaging and neurophysiology studies have demonstrated learning-dependent changes in functional brain connectivity in a range of tasks: visual perceptual learning (Baldassarre et al., 2012, Lewis et al., 2009), category learning (Antzoulatos & Miller, 2014), motor learning (Bassett et al., 2011, Ma et al., 2011, Sun et al., 2007), auditory learning (Ventura-Campos et al., 2013) and language learning (Veroude et al., 2010). However, most of this work has focused on reward-based learning that involves training with trial-by-trial feedback. Here, we show that learning temporal statistics may proceed without explicit trial-by-trial feedback and involve interactions between brain networks similar to those known to support reward-based learning (Alexander et al., 1986, Lawrence et al., 1998).

Finally, we considered whether the learning we observed occurred in an incidental manner or involved explicit knowledge of the underlying sequence structure. Previous studies have suggested that learning of regularities may occur implicitly in a range of tasks: visuomotor sequence learning (Nissen and Bullemer, 1987, Schwarb and Schumacher, 2012, Seger, 1994), artificial grammar learning (Reber, 1967), probabilistic category learning (Knowlton, Squire, & Gluck, 1994) and contextual cue learning (Chun & Jiang, 1998). This work has focused on implicit measures of sequence learning, such as familiarity judgments or reaction times. In contrast, our paradigm allows us to directly test whether exposure to temporal sequences facilitates the observers' ability to explicitly predict the identity of the next stimulus in a sequence. Although, our experimental design makes it unlikely that the participants memorized specific stimulus positions or the full sequences, debriefing the participants suggests that most extracted some high probability symbols or context-target combinations. Thus, it is possible that prolonged exposure to probabilistic structures (i.e., multiple sessions in contrast to single exposure sessions typically used in statistical learning studies) in combination with prediction judgments (Dale, Duran, & Morehead, 2012) may evoke some explicit knowledge of temporal structures, in contrast to implicit measures of anticipation typically used in statistical learning studies.

## 5 Conclusions

Our findings provide evidence that functional brain connectivity changes with learning in dissociable networks to support our ability to extract behaviorally-relevant statistics. This network connectivity relates to individual decision strategies when learning temporal structures. Our paradigm tested learning of structures that increased in context-length over time; thus, it does not allow us to dissociate learning time course from changes in sequence structure over time. In future work, it would be interesting to investigate the time course of learning temporal statistics using dynamic connectivity analysis that allows us to track changes in brain connectivity over time.

## Funding

This work was supported by grants to ZK from the Biotechnology and Biological Sciences Research Council [H012508], the Leverhulme Trust [RF-2011-378] and the [European Community's] Seventh Framework Programme [FP7/2007–2013] under agreement PITN-GA-2011-290011, AEW from the Wellcome Trust (095183/Z/10/Z) and the [European Community's] Seventh Framework Programme [FP7/2007–2013] under agreement PITN-GA-2012-316746, PT from Engineering and Physical Sciences Research Council [EP/L000296/1].
